# Effects of perioperative blood transfusion in gastric cancer patients undergoing gastrectomy: A systematic review and meta-analysis

**DOI:** 10.3389/fsurg.2022.1011005

**Published:** 2023-01-17

**Authors:** Wanqing Wang, Lulu Zhao, Penghui Niu, Xiaojie Zhang, Xiaoyi Luan, Dongbing Zhao, Yingtai Chen

**Affiliations:** National Cancer Center/National Clinical Research Center for Cancer/Cancer Hospital, Chinese Academy of Medical Sciences and Peking Union Medical College, Beijing, China

**Keywords:** gastric cancer, perioperative blood transfusions, overall survival, disease-free survival, disease-special survival, postoperative complications

## Abstract

**Background:**

The short-term and long-term effects of perioperative blood transfusion (PBT) on patients with gastric cancer are still intriguing. This systematic review and meta-analysis aimed to investigate the effects of blood transfusion on clinical outcomes in patients with gastric cancer undergoing gastrectomy.

**Methods:**

We searched PubMed, Web of Science, Embase, and The Cochrane Library on December 31th 2021. The main outcomes were overall survival (OS), disease-free survival (DFS), disease-specific survival (DFS), and postoperative complications. A fixed or random-effects model was used to calculate the hazard ratio (HR) with 95% confidence intervals (CIs).

**Results:**

Fifty-one studies with a total of 41,864 patients were included for this review and meta-analysis. Compared with patients who did not receive blood transfusions (NPBT), PBT was associated with worse 5-year OS (HR = 2.39 [95%CI: 2.00, 2.84]; *p* < 0.001; Multivariate HR = 1.43 [95%CI: 1.24, 1.63]; *p* < 0. 001), worse 5-year DFS (HR = 2.26 [95%CI: 1.68, 3.05]; *p* < 0.001; Multivariate HR = 1.45 [95%CI: 1.16, 1.82]; *p* < 0. 001), and worse 5-year DSS (HR = 2. 23 [95%CI: 1.35, 3.70]; *p* < 0.001; Multivariate HR = 1.24 [95%CI: 0.96, 1.60]; *p* < 0.001). Moreover, The PBT group showed a higher incidence of postoperative complications [OR = 2.30 (95%CI:1.78, 2. 97); *p* < 0.001] than that in the NPBT group, especially grade III-V complications, according to the Clavien-Dindo classification. [OR = 2.50 (95%CI:1.71, 3.63); *p* < 0.001].

**Conclusion:**

In patients who underwent gastrectomy, PBT was associated with negative survival effects (OS, DFS, DSS) and a higher incidence of perioperative complications. However, more research was expected to further explore the impact of PBT. Meanwhile, strict blood transfusion management should be implemented to minimize the use of PBT.

## 1. Introduction

Gastric cancer is an important cause of cancer-related death, ranking fifth for incidence and fourth for mortality worldwide (1). Radical surgery remains the only opportunity to cure gastric cancer (2). Surgical trauma and perioperative anemia often induce blood transfusions in gastric cancer patients but some studies had shown that there were potential risks that can be attributed to immunosuppression (3, 4). Although blood transfusion is widely used by surgeons, the appropriate transfusion strategy of perioperative blood transfusion (PBT) in gastric cancer patients undergoing gastrectomy is not clear.

Previous studies had shown that PBT had adverse effects on patients in different cancers, like prostate cancer (5), lung cancer (6), and hepatocellular cancer (7). But conclusions about the effect of blood transfusion on the prognosis of gastric cancer were contradictory. Some studies had reported a negative association between PBT and prognosis of gastric cancer (8–34), whereas others found no association (35–58). A previous meta-analysis (59) had reported a worse prognosis of gastric cancer patients with PBT but was limited by the small sample size and low credibility of the evidence. Results concentrated on PBT in gastric cancer patients needed to be further confirmed.

Therefore, the study conducted this systematic review and meta-analysis to identify and summarize existing evidence and attempted to define the relationships between PBT and short- or long-term prognosis in patients undergoing gastrectomy. The aim of this study is to provide guidance for clinical decision-making and further optimize the perioperative transfusion management of gastric cancer patients.

## 2. Methods

This meta-analysis was performed according to the PRISMA Checklist (60). The protocol has been registered in the International prospective register of systematic reviews database (Prospro number: CRD42022314772, https://www.crd.york.ac.uk/PROSPERO/).

### 2.1. Literature search and study selection

Two authors independently search the databases. The literature was systematically searched using Pubmed, Embase, The Cochrane Library, and Web of Science database on 31st December 2021 for studies published until December 2021. The search strategy was as follows: [(“Stomach Neoplasms” OR “neoplasm stomach” OR “Stomach Neoplasm” OR “neoplasms stomach” OR “Gastric Neoplasms” OR “Gastric Neoplasm” OR “neoplasm gastric” OR “neoplasms gastric” OR “Cancer of Stomach” OR “Stomach Cancers OR Gastric Cancer” OR “cancer gastric” OR “cancers gastric” OR “cancers gastric” OR “Stomach Cancer” OR “cancer stomach” OR “cancers stomach” OR “Cancer of the Stomach”) AND (“Blood Transfusion” OR “Blood Transfusions” OR “Transfusion, Blood” OR “Transfusions, Blood”)]. We also searched the reference lists of relevant studies and previous meta-analyses. Duplicates were excluded. After a preliminary review of the title and abstract, some articles investigating related to blood transfusion were included. The full text of including articles were screened for eligibility for data extraction.

### 2.2. Inclusion and exclusion criteria

Inclusion criteria were described as follows: (1) Studies evaluating the association between perioperative blood transfusion and prognosis of gastric cancer patients after gastrectomy; (2) At least including one of the outcomes: overall survival (OS), disease-free survival (DFS), disease-specific survival (DSS) and postoperative complications; (3) Human studies.

Exclusion criteria were described as follows: (1) Studies about benign gastric diseases, patients with double primary cancers, without surgical treatment or underwent palliative resection; (2) Studies not in English; (3) Data cannot be extracted; (4) Sample size less than 100; (5) Conference abstract or review was excluded.

Studies based on duplicate authors or centers were excluded and we chose the latest one for inclusion.

### 2.3. Data extraction

Two authors independently extracted the data from the included studies. For each article included in the meta-analysis, the following information was extracted: (1) Study information: name of the first author; year of publication; data collection method; location of the research; sample size; group selection; median follow-up and time of the last follow-up; (2) Characteristics of patients: age, gender, body mass index (BMI), hemoglobin (Hb), albumin (Alb), comorbidity, tumor size, depth of invasion, lymph node metastasis, stage, tumor location, histologic grade; (3) Surgery information: operation time, American Society of Anesthesiologists(ASA) score, gastrectomy type (total/subtotal, open/laparoscopic), splenectomy, estimated blood loss (EBL), PBT trigger, the quantity of PBT, time of PBT, chemotherapy. (4) Outcomes: OS, DFS, DSS, postoperative complications.

The multivariable HRs with 95% CI for OS, DFS, DSS, and survival data under different stages of patients were extracted if available. The assessment of stage and lymph-node metastasis were based on the American Joint Committee on Cancer (AJCC) staging system (61–64).

### 2.4. Quality assessment

The quality of included studies was assessed by two dependent reviewers using Newcastle-Ottawa Scale (NOS) (65). The literature quality was evaluated from three dimensions: group selection, comparability, and outcomes for cohort studies. The NOS contained eight items and ranged from zero up to nine stars.

### 2.5. Statistical analysis

Effects were expressed as weighted mean difference (WMD) with a corresponding 95% confidence interval (CI) for continuous variables and odds ratio (OR) with a corresponding 95% CI for categorical variables (66).

Heterogeneity was tested using the Chi-square test based on the Cochran Q statistic and *I*^2^ metric, and subgroup analyses and a meta-regression model were used to explore sources of heterogeneity.

Heterogeneity between studies was assessed by the Chi-square test and *I*^2^ tests. *I*^2^ values greater than 50% indicated significant heterogeneity (67). In the case of *I*^2^ > 50%, the summary HR and the accompanying 95% CI were calculated with a random-effects model, otherwise, a fixed-effects model was used.

We used forest plots to aggregate the HRs of outcomes from individual studies and funnel plots to examine the bias. We stratified OS data by G. location, average age, publication year, gender, estimated blood loss, transfusion rate, preoperative Hb, stage, transfusion trigger or transfusion quantity. Sensitivity analyses were conducted by removing individual studies in turns. Subgroup analyses and sensitivity analyses were used to analyze sources of significant heterogeneity.

The meta-analysis was performed by Review Manager (RevMan v.5.4) and R (v.4.1.0 x64) software. *P* value < 0. 05 was considered significant statistically.

## 3. Results

### 3.1. Selected studies

**Figure 1:**
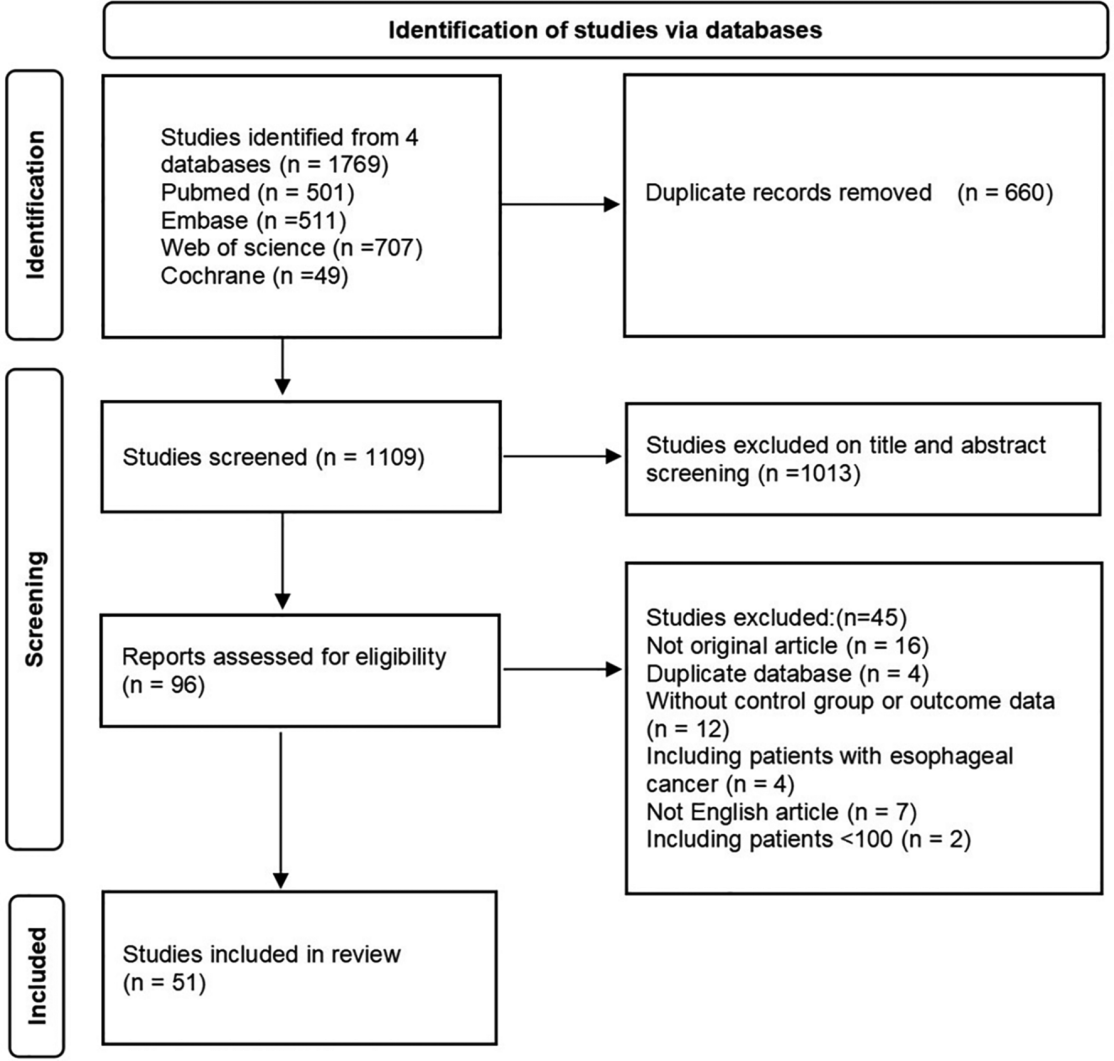
Flow chart of the study screening for this analysis.

A total of 1,769 articles were retrieved by searching electronic databases (Pubmed, Web of Science, Embase, and Cochrane). After the duplicates were differentiated and excluded, there were 1,109 articles remaining. We excluded the studies which were conference abstracts, non-English articles, duplicate databases, or centers by screening the title and abstract and excluded the studies that could not be extracted valid information. Finally, 51 studies (8–58) published from 1987 to 2021 that fulfilled the inclusion criteria were included. Figure 1 showed the flow chart of the search results. The reasons for excluding studies in the screening stage were shown in Table S3.

### 3.2. Characteristics of the patients and studies

A total of 41,864 patients were included in this meta-analysis, which involved 10,475 patients (25%) with PBT and 31,389 patients (75%) who did not receive perioperative blood transfusion (NPBT). The follow-up period ranged from 12 to 180 months, and the median was 56.2 months. The PBT rate of studies ranged from 3% to 74%. Definition of PBT was reported in 27 studies. The characteristics of these studies and patients were presented in Supplementary Tables S1, S2.

**Table 1:** Analysis of clinicopathological characteristics between the PBT group and NPBT group.

Group	Included studies	Included patients	*I* ^2^	Effect Model	OR/WMD	95%CI	*P*
Female	26	19,011	46%	Fixed	1.02	[0.95, 1.10]	0.51
Age	15	9,942	86%	Random	3.36	[2.14, 4.57]	<0.001
BMI	7	5,056	1%	Fixed	−0.14	[−0.43, 0.14]	0.33
Pre Alb	5	5,474	70%	Random	−0.36	[−0.42, −0.30]	<0.001
Comorbidity	5	3,237	0%	Fixed	1.25	[1.02, 1.53]	0.03
Preoperative anemia	3	626	0%	Fixed	10.83	[7.23, 16.21]	<0.001
Preoperative Hb	14	8,626	98%	Random	−2.19	[−3.02, −1.36]	<0.001
Depth of invasion							
Tis	3	998	0%	Fixed	0.81	[0.44, 1.51]	0.51
T1	16	10,710	74%	Random	0.41	[0.31, 0.53]	<0.001
T2	17	11,177	75%	Random	0.70	[0.54, 0.90]	0.005
T3	17	11,177	83%	Random	1.43	[1.09, 1.87]	0.009
T4	15	10,547	94%	Random	2.57	[1.44, 4.60]	0.001
Lymph-node metastasis	** **			Random			
N0	14	10,162	68%	Random	0.62	[0.52, 0.74]	<0.001
N1	13	9,486	74%	Random	1.03	[0.82, 1.30]	0.80
N2	13	9,486	64%	Random	1.49	[1.20, 1.86]	<0.001
N3	10	8,631	55%	Random	1.75	[1.41, 2.18]	<0.001
pTNM stage							
I	21	13,010	90%	Random	0.35	[0.25, 0.49]	<0.001
II	23	13,727	58%	Random	1.05	[0.90, 1.23]	0.55
III	24	14,926	61%	Random	1.89	[1.65, 2.18]	<0.001
IV	11	6,945	83%	Random	2.96	[1.78, 4.92]	<0.001
Tumor size	5	3,695	77%	Random	1.32	[0.90, 1.75]	<0.001
Tumor size > 5 cm	5	3,218	20%	Fixed	3.00	[2.54, 3.55]	<0.001
Tumor location							
Upper	15	10,407	80%	Random	1.54	[1.16, 2.04]	0.003
Middle	16	10,570	73%	Random	1.02	[0.84, 1.24]	0.84
Low	16	10,570	52%	Random	0.72	[0.63, 0.82]	<0.001
All stomach	8	5,841	41%	Fixed	2.27	[1.53, 3.36]	<0.001
Histologic grading							
Well/moderate	17	9,913	75%	Random	0.97	[0.80, 1.18]	0.74
Poor/undifferentiate	17	9,913	75%	Random	1.02	[0.93, 1.12]	0.67
Adjuvant chemotherapy	14	11,287	89%	Random	1.01	[0.73, 1.41]	0.93
ASA score > 2	4	4,061	30%	Fixed	1.91	[1.58, 2.32]	<0.001
EBL	9	5,180	98%	Random	216.1	[136.24, 295.96]	<0.001
Operation time	8	4,335	89%	Random	31.51	[17.64, 45.38]	<0.001
Type of gastrectomy							
Total	19	14,989	87%	Random	1.59	[1.24, 2.04]	<0.001
Subtotal	19	15,006	88%	Random	0.64	[0.50, 0.83]	<0.001
Open-gastrectomy	6	3,801	51%	Random	2.46	[1.65, 3.67]	<0.001
Lap-gastrectomy	6	3,801	51%	Random	0.41	[0.27, 0.61]	<0.001
Extended surgery							
Splenectomy	11	9,324	89%	Random	2.38	[1.56, 3.64]	<0.001
Multiple organ resection	14	11,993	92%	Random	2.33	[1.55, 3.52]	<0.001
Hospital stay time	4	3,971	36%	Fixed	1.26	[0.63, 1.89]	<0.001

BMI, body mass index; Pre, preoperative; Alb, albumin; Hb, hemoglobin; ASA, American society of anesthesiologists; EBL, estimated blood loss.

15 studies compared the age of patients and compared with the NPBT group, PBT group was older [OR: 3.36, 95%CI: (2.14, 4.57)]. 17 studies presented the preoperative Hb or anemia data, and we found patients with transfusion had a lower preoperative Hb level [OR: −2.19, 95%CI: (−3.02, −1.36)] or higher prevalence of preoperative anemia [OR: 10.83, 95%CI: (7.23, 16.21)]. Besides, PBT group have higher rate of comorbidity [OR: 1.25, 95%CI: (1.02, 1.53)] and lower preoperative albumin level [OR: −0.36, 95%CI: (−0.42, −0.30)]. There were no significant differences in different gender and BMI.

According to the TNM stage system (61–64), data from eligible studies showed that pathological stages of PBT group were more likely to be stage III[OR: 1.89, 95%CI: (1.65, 2.18)] and stage IV [OR: 2.57, 95%CI: (1.44,4.60)]. 17 studies reported the depth of invasion of tumor and 14 studies reported the lymph node metastasis. PBT group had a higher ratio of T3 [OR: 1.43, 95%CI: (1.09, 1.87)], T4 [OR: 2.57, 95%CI: (1.44, 4.60)], N2[OR: 1.49, 95%CI: (1.20, 1.86)], and N3[OR: 1.75, 95%CI: (1.41, 2.18)]. Differences of tumor location (upper location: OR: 1.54, 95%CI: [1.16, 2.04]; all stomach: OR: 2.27, 95%CI: [1.53, 3.36]) and tumor size (larger tumor size: OR: 1.32, 95%CI: [0.90, 1.75]; tumor size > 5 cm: OR:3.00, 95%CI: [2.54, 3.55]) were also found. However, as for histological differentiation, there was no significant difference between the two groups.

More than two thirds of studies presented the operation data. PBT group had a higher rate of conversion to open surgery [OR: 2.46, 95%CI: (1.65, 3.67)], total gastrectomy [OR: 1.59, 95%CI: (1.24, 2.04)] and multi-organ resection [OR: 2.33, 95%CI: (1.55, 3.52)], especially splenectomy [OR: 2.38, 95%CI: (1.56, 3.64)]. Besides, patients with PBT had higher ASA scores [ASA > 2: OR: 1.91, 95%CI: (1.58, 2.32)], greater EBL [OR: 216.1, 95%CI: (136.24, 295.96)] and longer hospital stay time [OR: 1.26, 95%CI: (0.63,1.89)] when compared with patients without PBT (Table 1).

### 3.3. Postoperative complications

**Figure 2:**
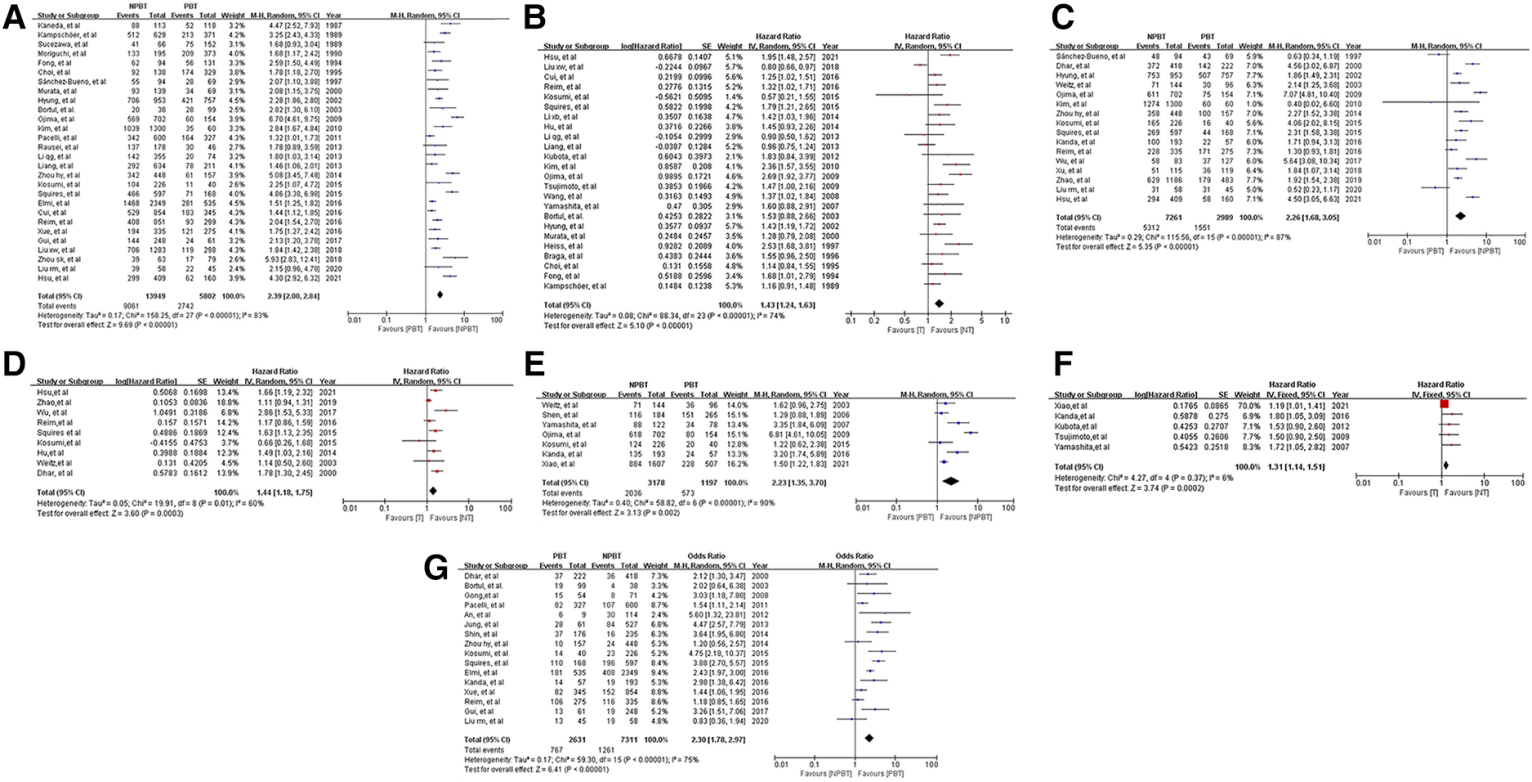
Forest plots and results of the meta-analysis of studies. (**A**) Forest plot of overall survival (OS) based on univariate results of studies. (**B**) Forest plot of OS based on multivariate results of studies. (**C**) Forest plot of disease-free survival (DFS) based on univariate results of studies. (**D**) Forest plot of DFS based on multivariate results of studies. (**E**) Forest plot of disease-specific survival (DSS) based on univariate results of studies. (**F**) Forest plot of DSS based on multivariate results of studies. (**G**) Forest plot of postoperative complications based on results of studies.

16 studies with 9,942 patients showed postoperative complications after gastrectomy. The OR of postoperative complications was 2.30 [95%CI: (1.78, 2.97)]. According to the Clavien-Dindo grade (68), the PBT group had a higher incidence rate of grade III-V complications [OR: 2.50, 95%CI: (1.71, 3.63); *p* < 0. 01], whereas no significant difference was seen in grade I-II [OR: 1.12, 95%CI: (0.63, 2.00); *p* = 0.69]. (Table 2) The forest plot and funnel plot were shown in Figure 2G, Supplementary Figure S1G.

**Table 2:** Analysis of postoperative complications between the PBT group and not-PBT group.

Outcome	Included studies	Included patients	*I* ^2^	Effect Model	OR	95%CI	*P*
Postoperative complications	16	9,942	75%	Random	2.30	[1.78, 2.97]	<0.001
Clavien-Dindo grade							
Grade I–II	10	7,918	90%	Random	1.12	[0.63, 2.00]	0.69
Grade III–V	6	5,371	79%	Random	2.5	[1.71, 3.63]	<0.001

### 3.4. Long-term outcomes

#### 3.4.1. Overall survival

36 studies reported data on OS. Data on 5-year OS was available from 28 studies and HRs after multivariable analyses were extracted from 24 studies. The total number of enrolled patients was 25,122, with individual samples ranging from 103 to 2,884 (median 699). The HR of 5-year OS was 2.39 [95% CI: (2.00, 2.84), *P* < 0.01] and the summary of the multivariable HR was 1.43 [95% CI: (1.24, 1.63)]. Measure of heterogeneity indicates a high degree of variability about 5-year OS (HR: *I*^2^ = 83%, *P* < 0.01; multivariable HR: *I*^2^ = 74%, *P* < 0.01). The random-effects model was used to obtain estimates. The forest plots of OS were shown in Figures 2A,B.

A stratified analysis of OS was performed and the results were shown in Table 3. Publication years (before or after 2010), NOS score (≤7 stars or >7 stars), geographical location (west or east), average age (≤60 or >60), EBL (≤500 ml or >500 ml), PBT trigger (Hb < 7 g/L or Hb < 8 g/L), PBT rate (≤40% or >40%) and quantity (4U ≤ 50% or 4U > 50%) did not change the outcome significantly, which showed the result was robust.

**Table 3:** Stratified meta-analysis of overall survival comparison between the PBT group and NPBT group.

Subgroup	Included studies	Included patients	*I* ^2^	Effect Model	HR	95%CI	*P*
G.location							
West	8	6,475	83%	Random	2.15	[1.55, 2.97]	<0.001
East	20	13,276	82%	Random	2.49	[2.02, 3.07]	<0.001
Average age							
≤60	4	2,890	0%	Random	2.07	[1.77, 2.41]	<0.001
>60	8	6,163	89%	Random	2.8	[1.84, 4.28]	<0.001
Year							
1987-2010	11	5,783	77%	Random	2.61	[2.00, 3.41]	<0.001
2011-2022	17	13,968	84%	Random	2.26	[1.80, 2.82]	<0.001
NOS score							
>7	12	8,344	74%	Random	2.30	[1.86, 2.84]	<0.001
≤7	16	11,407	95%	Random	2.04	[1.34, 3.12]	<0.001
EBL							
≤500 ml	4	2,216	89%	Random	2.41	[1.21, 4.80]	0.01
>500 ml	4	2,300	92%	Random	2.59	[1.27, 5.27]	0.009
PBT rate							
≤40%	17	14,637	89%	Random	2.39	[1.86, 3.09]	<0.001
>40%	11	5,114	44%	Random	2.29	[1.90, 2.76]	<0.001
Preoperative Hb							
≤11 g/L	6	3,110	85%	Random	2.06	[1.18, 3.63]	<0.001
>11 g/L	5	4,622	69%	Random	1.66	[1.30, 2.12]	0.01
PBT trigger							
Hb < 7 g/L	4	3,259	91%	Random	2.05	[1.22, 3.44]	<0.001
Hb < 8 g/L	3	2,981	70%	Random	4.68	[3.02, 7.27]	0.03
PBT quantity							
4U ≤ 50%	3	3,247	80%	Random	1.75	[1.25, 2.47]	0.001
4U > 50%	4	3,184	93%	Random	4.29	[2.08, 8.85]	<0.001

G. locations, geographical location; NOS, New castle Ottawa scale; EBL, estimated blood loss; PBT, perioperative blood transfusion; Hb, hemoglobin.

Sensitivity analysis, which explored the effect on overall results by sequentially omitting individual studies, and a baujat plot was conducted to explore the source of heterogeneity between studies. (Supplementary Figure S2). 6 studies (9, 13, 21, 32, 42, 50) might be the main reason for the high heterogeneity. The funnel plot showed obvious asymmetry and publication bias was detected (Supplementary Figures S1A,B).

Moreover, further survival analyses were performed under different tumor stages according to the pTNM stage system. There were 7 studies, 8 studies, and 6 studies that showed survival rates between different groups at stages I, II, and III respectively. Compared to the NPBT patients, the PBT group was associated with lower 1-, 2-, 3-year OS at stages I, II, and III and lower 5-year OS at stage I [HR:2.54, 95%CI: (1.46, 4.44); *p* < 0.001;], III [HR:1.62, 95%CI: (1.38, 1.92); *p* < 0.001] whereas there was no significant difference in 5-year OS among stage II patients [HR:1.46, 95%CI: (0.92, 2.32); *p* = 0.11]. (Table 4).

**Table 4:** Survival outcomes of patients between the PBT group and Not-PBT group.

Outcome	Included studies	Included patients	*I* ^2^	Effect Model	HR	95%CI	*P*
OS							
1y-OS	23	15,616	79%	Random	2.28	[1.75, 2.97]	<0.001
2y-OS	23	15,616	85%	Random	2.04	[1.62, 2.57]	<0.001
3y-OS	23	15,616	87%	Random	2.02	[1.61, 2.53]	<0.001
5y-OS	28	19,751	83%	Random	2.39	[2.00, 2.84]	<0.001
10y-OS	5	4,527	91%	Random	1.56	[0.98, 2.46]	0.06
OS-Multivariate HR	24	13,898	74%	Random	1.43	[1.24, 1.63]	<0.001
Stage Ι-OS							
1-year OS	7	2,781	37%	Fixed	2.38	[1.46, 3.88]	<0.001
2-year OS	7	2,781	56%	Random	2.50	[1.35, 4.63]	0.004
3-year OS	7	2,781	33%	Fixed	2.66	[1.94, 3.64]	<0.001
5-year OS	7	2,781	69%	Random	2.54	[1.46, 4.44]	0.001
Stage II-OS							
1-year OS	6	1,152	0%	Fixed	2.33	[1.42, 3.84]	<0.001
2-year OS	6	1,152	0%	Fixed	2.13	[1.50, 3.01]	<0.001
3-year OS	6	1,152	0%	Fixed	1.83	[1.35, 2.47]	<0.001
5-year OS	6	1,152	55%	Random	1.46	[0.92, 2.32]	0.11
Stage III-OS							
1-year OS	8	2,994	38%	Fixed	1.93	[1.56, 2.39]	<0.001
2-year OS	8	2,994	22%	Fixed	1.71	[1.45, 2.03]	<0.001
3-year OS	8	2,994	40%	Fixed	1.70	[1.45, 2.00]	<0.001
5-year OS	8	2,994	0%	Fixed	1.62	[1.38, 1.92]	<0.001
DFS							
1y-DFS	11	6,178	81%	Random	2.66	[1.79, 3.96]	<0.001
2y-DFS	7	4,313	88%	Random	2.12	[1.43, 3.13]	<0.001
3y-DFS	12	7,195	91%	Random	2.23	[1.44, 3.46]	<0.001
5y-DFS	16	10,250	87%	Random	2.26	[1.68, 3.05]	<0.001
10y-DFS	4	3,088	96%	Random	1.47	[0.59, 3.70]	0.41
DFS-Multivariate HR	9	7,698	60%	Random	1.44	[1.18, 1.75]	<0.001
DSS							
1y-DSS	4	2,804	54%	Random	1.56	[0.94, 2.57]	0.08
2y-DSS	4	2,804	73%	Random	2.16	[1.34, 3.49]	0.002
3y-DSS	4	2,804	81%	Random	2.45	[1.43, 4.19]	0.001
5y-DSS	7	4,375	90%	Random	2.23	[1.35, 3.70]	0.002
DSS- Multivariate HR	6	5,153	0%	Fixed	1.35	[1.21, 1.51]	<0.001

OS, overall suivival; DFS, disease-free survival; DSS, disease-specific survival; HR, hazard ratio.

#### 3.4.2. Disease-free survival

17 studies reported data on DFS. Data on 5-year DFS were available from 16 studies and HRs after multivariable analyses were extracted from 9 studies. The 5-year DFS was lower in patients with PBT than NPBT patients. (HR = 2.26, 95% CI: [1.68, 3.05]; multivariable HR = 1.44, 95% CI: [1.18, 1.75]). *I*(2) as shown in Table 4. The funnel plot showed obvious asymmetry (Supplementary Figures S1C,D). The forest plots of DFS were shown in Figures 2C,D.

#### 3.4.3. Disease-specific survival

9 studies reported data on DSS. Data on 5-year DSS were available from 7 studies and HRs after multivariable analyses were extracted from 6 studies. The 5-year DSS was lower in patients with PBT than NPBT patients. (HR = 2.23, 95% CI: [1.35, 3.70]; multivariable HR = 1.35, 95% CI: [1.21, 1.51]). *I*^2^ as shown in Table 4. The funnel plot showed obvious asymmetry (Supplementary Figures S1E,F). The forest plots of DFS were shown in Figures 2E,F.

## 4. Discussion

To date, the effects of PBT on the prognosis of gastric cancer patients undergoing gastrectomy were still controversial, and consensus had not yet been reached finally. The review and meta-analysis involved 51 studies with 41,864 gastric cancer patients. To our best knowledge, this analysis represented the largest assessment of current research that targeted the impact of PBT on the long- and short-term outcomes. A primary finding was that PBT was associated with worse prognosis than the NPBT group.

Specifically, the results of the meta-analysis showed that PBT was associated with worse 1-,2-,3- and 5-year OS (82% vs. 91%; 66% vs. 80%; 57% vs. 72%; 47% vs. 65%), DFS (76% vs. 88%; 61% vs. 76%; 53% vs. 74%; 52% vs. 73%), and DSS (86% vs. 89%; 64% vs. 74%; 53% vs. 66%; 48% vs. 64%). The results were similar to the conclusions of previous research (59, 69–72). Similar results were found in other meta-analyses of other solid cancer, including colorectal cancer (73, 74), hepatic cancer (75), esophageal cancer (76, 77), and pancreatic cancer (78). Further, we conducted stratified analysis and sensitivity analysis of OS and the results were consistent and credible. The mechanism could be partially attributed to the suppression of the immune system induced by blood transfusion (79). Firstly, some studies showed that the patients with previous blood transfusions experienced changes in the immune system (80–82) involving inhibition of T cells and alteration in T cell subsets (83). Secondly, transfusion could trigger a series of a cascade of the immune system, including inhibition of the immunoregulatory cytokine IL-2, and the release of immunosuppressive prostaglandins 3. Besides, blood transfusion could induce transfusion-related immunomodulation (TRIM), further inhibits the function of macrophages and monocytes (84), and might lead to the decline of immune surveillance and enhance the potential for tumor growth and cellular metastasis.

Significant differences in the clinicopathological characteristics were found between the PBT group and NPBT group, which were consistent with previous studies 69. Compared with the NPBT group, the PBT group was more likely to be anemic and had lower Hb levels. Previous studies had shown that preoperative anemia was a powerful predictor of the need for blood transfusion and independently associated with an increased risk of mortality in patients undergoing surgery, even to a mild degree (85, 86). Besides, the PBT group had more advanced tumor stages, more open surgery or total gastrectomy, and more EBL. Intraoperative blood transfusion was more likely to result from the complicated operation, especially large EBL (87). In addition, patients with transfusion were older and had more comorbidities, which might also be one of the important reasons for the poor prognosis in the PBT group.

Moreover, our findings showed that the PBT group had a higher postoperative complication rate. After grading the complications according to the Clavien-Dindo grade system, PBT was particularly related to grade III-V complications, but there was no significant difference in grade I-II when compared with the NPBT group. To date, the mechanisms that targeted the association between PBT and postoperative complications were unclear. Previous studies showed that the clinicopathological features of the patients in two groups might independently influence the postoperative complications (44, 47, 88). Compared with the NPBT group, patients with PBT were prone to suffer from more surgical trauma and had less tolerance for surgery because of their poor clinical condition. These clinicopathological factors, including old, advanced tumor stage, and complicated type of surgery, might be also associated with postoperative complications (89–91). Relevant mechanisms were expected to be demonstrated further.

Strengths and limitations should be considered when interpreting the study results. In our literature review, we retrieved 3 meta-analysis and systematic reviews related to the effect of perioperative blood transfusion in gastric cancer patients published in 2015 (70, 71) or 2018 (59). These studies were limited by the small number of articles included, univariate analysis or high heterogeneity. In this meta-analysis, the number of studies included was the largest, and adopting the multivariable HR to overcome the potential bias, which made the results more reliable. Besides, we focused on the relationship between the PBT and OS, DFS, DSS, and postoperative complications of gastric cancer patients, and found the relationship between PBT and severe postoperative complications. Nevertheless, there were some limitations to this meta-analysis. For obvious ethical reasons, no randomized controlled trial (RCT) was searched and included in this meta-analysis. The heterogeneity of some results was high in this meta-analysis, which might be attributed to the wide span of publication years, different transfusion triggers, and lacking PBT guideline. In addition, few studies presented the data on the amount and components of blood transfusion and the time of PBT, this meta-analysis failed to conduct further research. More research was expected to explore the role of PBT and the appropriate PBT management strategy.

## 5. Conclusions

In conclusion, PBT was associated with adverse effects on the prognosis of gastric cancer patients undergoing gastrectomy, including OS, DFS, and DSS in this meta-analysis. In addition, PBT had a negative impact on postoperative complications in gastric cancer patients, especially grade III-V complications. The quality of the evidence was not high and bias were detected, which might lead to more significant results. But these results indicated that strict patient blood management strategies aimed at minimizing PBT were necessary. Future studies should be performed to further define the role of PBT and explore the guideline of PBT in gastric cancer patients.
