# Supplementary figures and images for: Effects of perioperative blood transfusion in gastric cancer patients undergoing gastrectomy: A systematic review and meta-analysis
Source: Front Surg. 2023 Jan 17;9:1011005. doi: 10.3389/fsurg.2022.1011005 (PMC9887286; doi:10.3389/fsurg.2022.1011005)

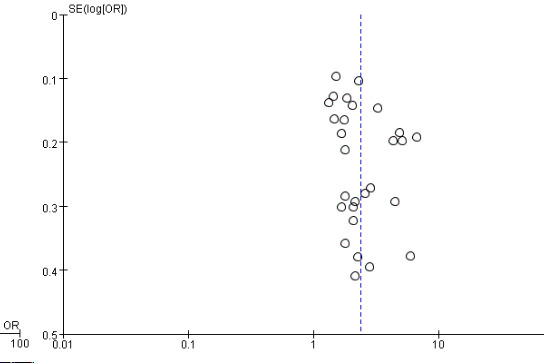

Supplement: Supplementary file 1 [file SupplementaryImage1.jpg]

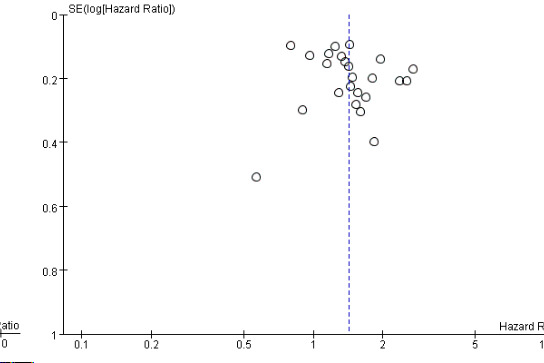

Supplement: Supplementary file 2 [file SupplementaryImage2.jpg]

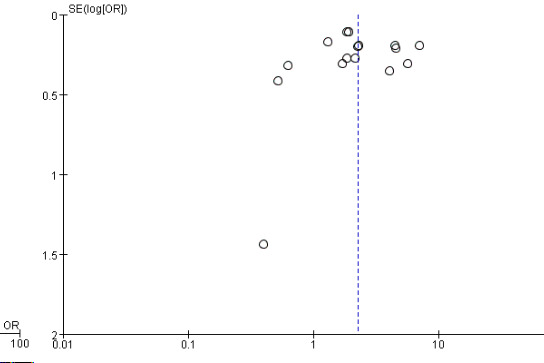

Supplement: Supplementary file 3 [file SupplementaryImage3.jpg]

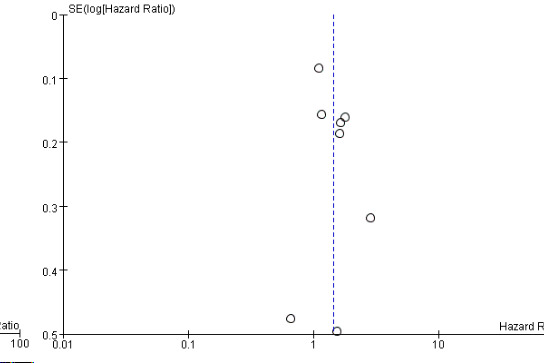

Supplement: Supplementary file 4 [file SupplementaryImage4.jpg]

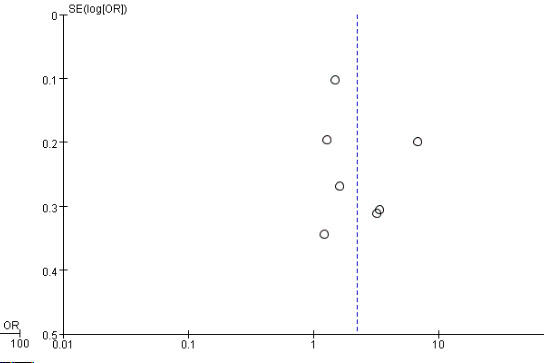

Supplement: Supplementary file 5 [file SupplementaryImage5.jpg]

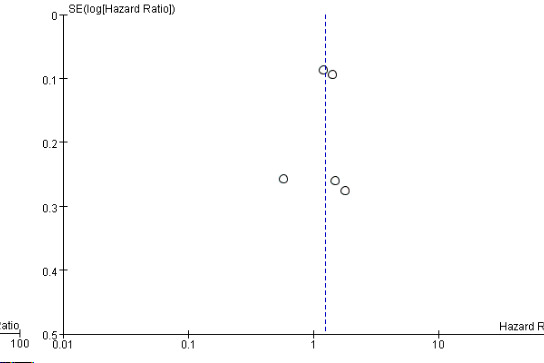

Supplement: Supplementary file 6 [file SupplementaryImage6.jpg]

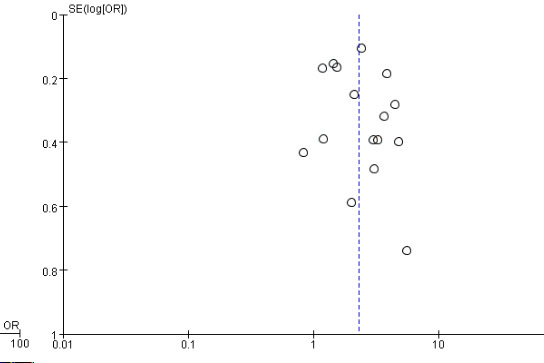

Supplement: Supplementary file 7 [file SupplementaryImage7.jpg]

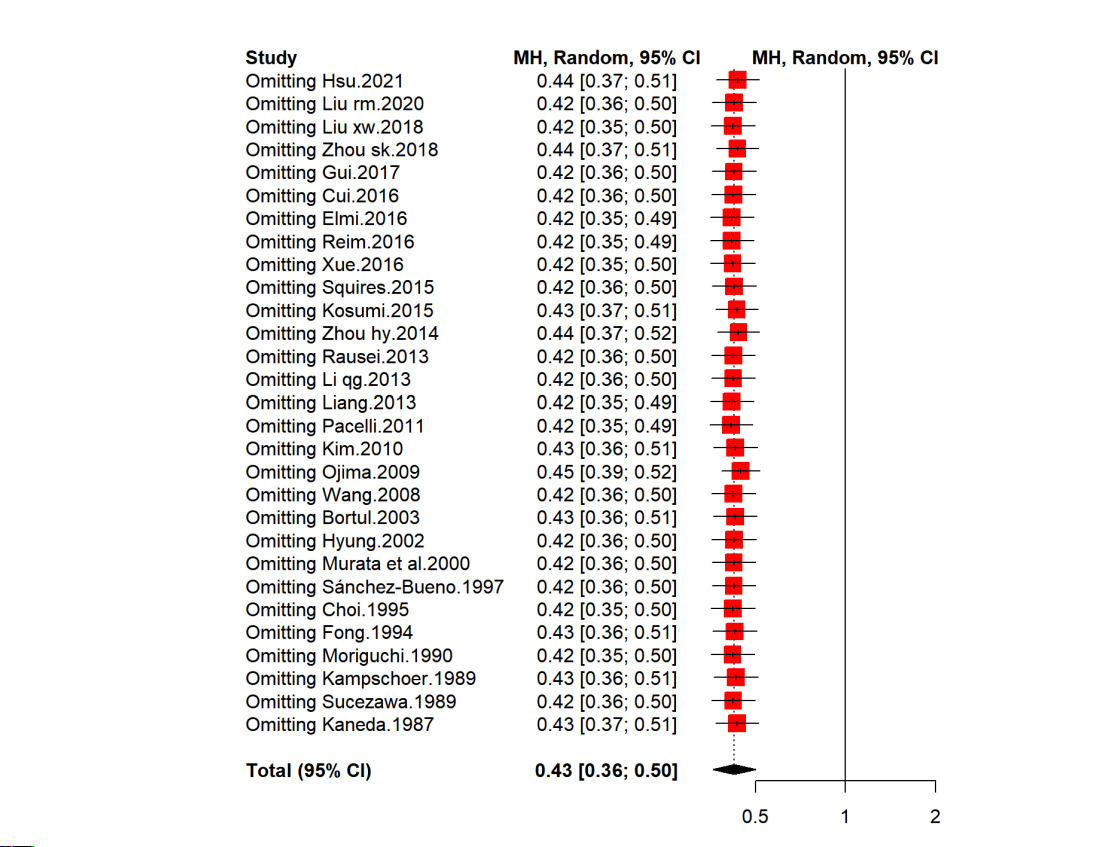

Supplement: Supplementary file 8 [file SupplementaryImage8.jpg]

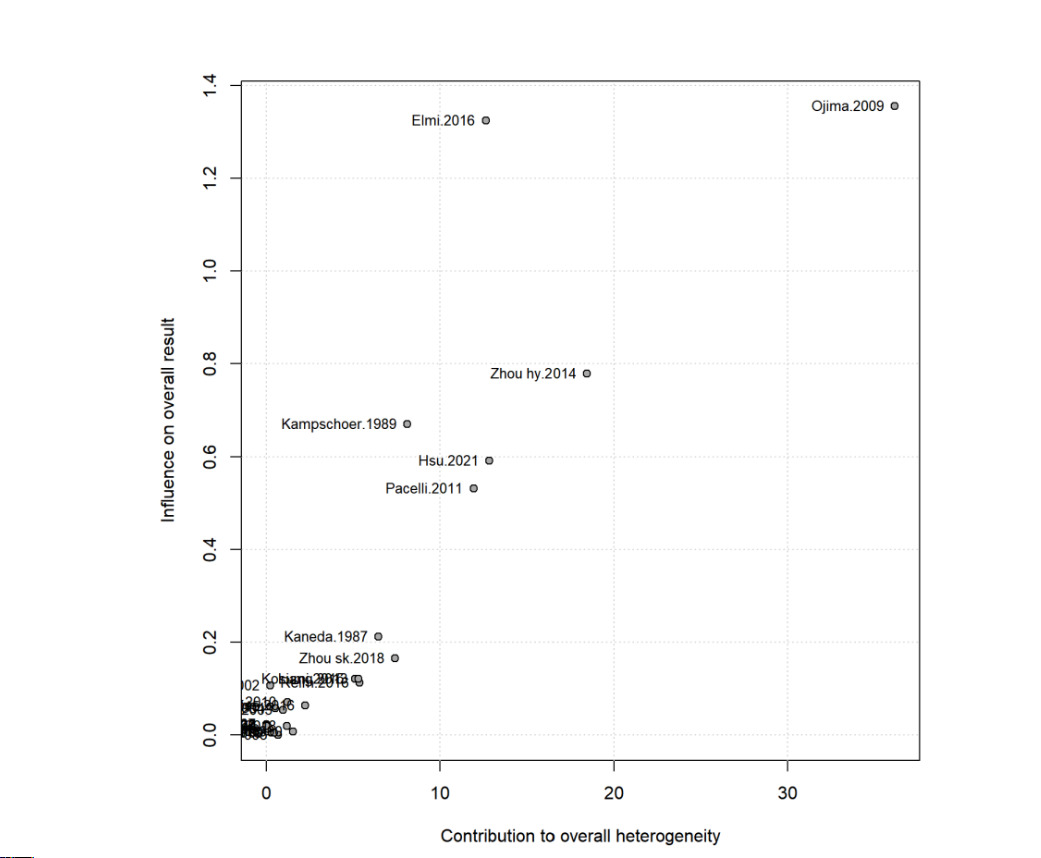

Supplement: Supplementary file 9 [file SupplementaryImage9.jpg]
