# Supplementary material for: Effects of perioperative blood transfusion in gastric cancer patients undergoing gastrectomy: A systematic review and meta-analysis
Source: Front Surg. 2023 Jan 17;9:1011005. doi: 10.3389/fsurg.2022.1011005 (PMC9887286; doi:10.3389/fsurg.2022.1011005)
Supplement: Supplementary file 13 [file TableS4.docx]

| **Section and Topic** | **Item #** | **Checklist item** | **Location where item is reported** |
| --- | --- | --- | --- |
| **TITLE** | | |  |
| Title | 1 | **Effects of perioperative blood transfusion in gastric cancer patients undergoing gastrectomy: A systematic review and a meta-analysis** |  |
| **ABSTRACT** | | |  |
| Abstract | 2 | **Abstract**  **Background**: The short-term and long-term effects of perioperative blood transfusion (PBT) on patients with gastric cancer are still intriguing. This systematic review and meta-analysis aimed to investigate the effects of blood transfusion on clinical outcomes in patients with gastric cancer undergoing gastrectomy.  **Methods**: We searched PubMed, Web of Science, Embase, and The Cochrane Library on December 31th 2021. The main outcomes were overall survival (OS), disease-free survival (DFS), disease-specific survival (DFS), and postoperative complications. A fixed or random-effects model was used to calculate the hazard ratio (HR) with 95% confidence intervals (CIs).  **Results**: Fifty-one studies with a total of 41864 patients were included for this review and meta-analysis. Compared with patients who did not receive blood transfusions (NPBT), PBT was associated with worse 5-year OS (HR=2.39 [95%CI: 2.00, 2.84]; p<0.001; Multivariate HR=1.43 [95%CI: 1.24, 1.63]; p<0. 001), worse 5-year DFS (HR = 2.26 [95%CI: 1.68, 3.05]; p<0.001; Multivariate HR=1.45 [95%CI: 1.16, 1.82]; p<0. 001), and worse 5-year DSS (HR = 2. 23 [95%CI: 1.35, 3.70]; p<0.001; Multivariate HR=1.24 [95%CI: 0.96, 1.60]; p<0.001). Moreover, The PBT group showed a higher incidence of postoperative complications (OR=2.30 [95%CI:1.78, 2. 97]; p<0.001) than that in the NPBT group, especially grade Ⅲ-Ⅴ complications, according to the Clavien-Dindo classification. (OR=2.50 [95%CI:1.71, 3.63]; p<0.001).  **Conclusion**: In patients who underwent gastrectomy, PBT was associated with negative survival effects (OS, DFS, DSS) and a higher incidence of perioperative complications. However, more research was expected to further explore the impact of PBT. Meanwhile, strict blood transfusion management should be implemented to minimize the use of PBT. |  |
| **INTRODUCTION** | | |  |
| Rationale | 3 | Gastric cancer is an important cause of cancer-related death, ranking fifth for incidence and fourth for mortality worldwide. Radical surgery remains the only opportunity to cure gastric cancer. Surgical trauma and perioperative anemia often induce blood transfusions but the appropriate transfusion strategy of perioperative blood transfusion (PBT) in gastric cancer patients undergoing gastrectomy is not clear.  Conclusions about the effect of blood transfusion on the prognosis of gastric cancer were contradictory. Some studies had reported a negative association between PBT and prognosis of gastric cancer, whereas others found no association. A previous meta-analysis had reported a worse prognosis of PBT but was limited by the small sample size and low credibility of the evidence. Results concentrated on PBT in gastric cancer patients needed to be further confirmed. |  |
| Objectives | 4 | The study conducted this systematic review and meta-analysis to identify and summarize existing evidence and attempted to define the relationships between PBT and short- or long-term prognosis in patients undergoing gastrectomy. |  |
| **METHODS** | | |  |
| Eligibility criteria | 5 | Inclusion criteria were described as follows: (1) Studies evaluating the association between perioperative blood transfusion and prognosis of gastric cancer patients after gastrectomy;(2) At least including one of the outcomes: overall survival (OS), disease-free survival (DFS), disease-specific survival (DFS) and postoperative complications;(3) Human studies.  Exclusion criteria were described as follows: (1) Studies about benign gastric diseases, patients with double primary cancers, without surgical treatment or underwent palliative resection;(2) Studies not in English;(3) Data cannot be extracted;(4) Sample size less than 100;(5) Conference abstract or review was excluded.  Studies based on duplicate authors or centers were excluded and we chose the latest one for inclusion. |  |
| Information sources | 6 | The literature was systematically searched using Pubmed, Embase, The Cochrane Library, and Web of Science database on 31st December 2021 for studies published until December 2021. |  |
| Search strategy | 7 | Two authors independently search the databases. The search strategy is as follows: (("Stomach Neoplasms" OR "neoplasm stomach" OR "Stomach Neoplasm" OR "neoplasms stomach" OR "Gastric Neoplasms" OR "Gastric Neoplasm" OR "neoplasm gastric" OR "neoplasms gastric" OR "Cancer of Stomach" OR "Stomach Cancers OR Gastric Cancer" OR "cancer gastric" OR "cancers gastric" OR "cancers gastric" OR "Stomach Cancer" OR "cancer stomach" OR "cancers stomach" OR "Cancer of the Stomach") AND ("Blood Transfusion" OR "Blood Transfusions" OR "Transfusion, Blood" OR "Transfusions, Blood")). |  |
| Selection process | 8 | Two authors independently screened titles and abstracts to identify articles for full-text examination. The reference lists of relevant studies and previous meta-analyses are also obtained. Duplicates were excluded. After a preliminary review of the title and abstract, some articles investigating related to blood transfusion were included. The full text of including articles was screened for eligibility for data extraction. |  |
| Data collection process | 9 | Two authors independently extracted the data from the included studies. The assessment of stage and lymph-node metastasis were based on the American Joint Committee on Cancer (AJCC) staging system. The multivariable HRs with 95% CI for OS, DFS, DSS, and survival data under different stages were extracted if available. |  |
| Data items | 10a | Outcome: overall-survival (OS), disease-specific survival (DFS), disease-specific survival (DSS), postoperative complications. |  |
|  | 10b | (1)Studies information: name of the first author; year of publication; data collection method; location of the research; sample size; group selection; median follow-up and time of the last follow-up; (2) Characteristics of patients: age, gender, body mass index (BMI), hemoglobin (Hb), albumin (Alb), comorbidity, tumor size, depth of invasion, lymph node metastasis, stage, tumor location, histologic grade; (3) Surgery information: operation time, American Society of Anesthesiologists(ASA) score, gastrectomy type (total/subtotal, open/laparoscopic), splenectomy, estimated blood loss (EBL), PBT trigger, the quantity of PBT, time of PBT, chemotherapy. |  |
| Study risk of bias assessment | 11 | The quality of included studies was assessed by two dependent reviewers using Newcastle-Ottawa Scale (NOS). The literature quality was evaluated from three dimensions: group selection, comparability, and outcomes for cohort studies. The NOS contained eight items and ranged from zero up to nine stars. |  |
| Effect measures | 12 | Effects were expressed as weighted mean difference (WMD) with a corresponding 95% confidence interval (CI) for continuous variables and hazard ratio (HR) with a corresponding 95% CI for categorical variables. |  |
| Synthesis methods | 13a |  |  |
|  | 13b |  |  |
|  | 13c |  |  |
|  | 13d | Heterogeneity between studies was assessed by the Chi-square test and *I*^2^ tests. *I*^2^ values greater than 50% indicated significant heterogeneity. In the case of *I*^2^>50%, the summary HR and the accompanying 95% CI were calculated with a random-effects model, otherwise, a fixed-effects model was used. |  |
|  | 13e | We used forest plots to aggregate the HRs from individual studies of outcomes and funnel plots to examine the bias. We performed subgroup analysis further to explore potential confounding influencing OS. We stratified OS data by G. location, average age, publication year, gender, estimated blood loss, transfusion rate, preoperative Hb, stage, and transfusion trigger transfusion quantity. Sensitivity analyses were used to detect publication bias by removing individual studies in turns and analyzing the effect on the OS to identify sources of significant heterogeneity. |  |
|  | 13f | Sensitivity analyses were used to detect publication bias by removing individual studies in turns and analyzing the effect on the OS to identify sources of significant heterogeneity. |  |
| Reporting bias assessment | 14 | A baujat plot was conducted to explore the source of heterogeneity between studies. |  |
| Certainty assessment | 15 | Stratified analysis and sensitivity analysis did not change the outcome significantly, which showed the result was robust. |  |
| **RESULTS** | | |  |
| Study selection | 16a | The selection flow diagram was shown in Figure 1. |  |
|  | 16b | The excluded and included reasons were shown in Table S2. |  |
| Study characteristics | 17 | A total of 1769 articles were retrieved by searching electronic databases (Pubmed, Web of Science, Embase, and Cochrane). After the duplicates were differentiated and excluded, there were 1109 articles remaining. We excluded the studies which were conference abstracts, non-English articles, duplicate databases, or centers by screening the title and abstract and excluded the studies that could not be extracted valid information. Finally, 51 studies published from 1987 to 2021 that fulfilled the inclusion criteria were included. |  |
| Risk of bias in studies | 18 | The NOS scores were shown in the Table S1. |  |
| Results of individual studies | 19 | Table S1 showed the characteristics of the included studies. And the forest plot was shown in the Figure 1. |  |
| Results of syntheses | 20a | A total of 41864 patients were included in this meta-analysis, which involved 10475 patients (25%) with PBT and 31389 patients (75%) who did not receive perioperative blood transfusion (NPBT). The follow-up period ranged from 12-180 months, and the median was 56. 2 months. The PBT rate of studies ranged from 3% to 74%. Definition of PBT was reported in 27 studies. The characteristics of these studies and patients were presented in Table S1 and Table 1. |  |
|  | 20b | 15 studies compared the age of patients and compared with the NPBT group, PBT group was older (OR: 3.36, 95%CI: [2.14, 4.57]). 17 studies presented the preoperative Hb or anemia data, and we found patients with transfusion had a lower preoperative Hb level (OR: -2.19, 95%CI: [-3.02, -1.36]) or higher prevalence of preoperative anemia (OR: 10.83, 95%CI: [7.23, 16.21]). Besides, PBT group have higher rate of comorbidity (OR: 1.25, 95%CI: [1.02, 1.53]) and lower preoperative albumin level (OR: -0.36, 95%CI: [-0.42, -0.30]). There were no significant differences in different gender and BMI.  According to the TNM stage system, data from eligible studies showed that pathological stages of PBT group were more likely to be stage Ⅲ(OR: 1.89, 95%CI: [1.65, 2.18]) and stage Ⅳ(OR: 2.57, 95%CI: [1.44,4.60]). 17 studies reported the depth of invasion of tumor and 14 studies reported the lymph node metastasis. PBT group had a higher ratio of T3 (OR: 1.43, 95%CI: [1.09, 1.87]), T4 (OR: 2.57, 95%CI: [1.44, 4.60]), N2(OR: 1.49, 95%CI: [1.20, 1.86]), and N3(OR: 1.75, 95%CI: [1.41, 2.18]). Differences of tumor location (upper location: OR: 1.54, 95%CI: [1.16, 2.04]; all stomach: OR: 2.27, 95%CI: [1.53, 3.36]) and tumor size (larger tumor size: OR: 1.32, 95%CI: [0.90, 1.75]; tumor size>5cm: OR:3.00, 95%CI: [2.54, 3.55]) were also found. However, as for histological differentiation, there was no significant difference between the two groups.  More than two thirds of studies presented the operation data. PBT group had a higher rate of conversion to open surgery (OR: 2.46, 95%CI: [1.65, 3.67]), total gastrectomy (OR: 1.59, 95%CI: [1.24, 2.04]) and multi-organ resection (OR: 2.33, 95%CI: [1.55, 3.52]), especially splenectomy (OR: 2.38, 95%CI: [1.56, 3.64]). Besides, patients with PBT had higher ASA scores (ASA>2: OR: 1.91, 95%CI: [1.58, 2.32]), greater EBL (OR: 216.1, 95%CI: [136.24, 295.96]) and longer hospital stay time (OR: 1.26, 95%CI: [0.63,1.89]) when compared with patients without PBT. (Table 1)  **Postoperative complications**  16 studies with 9942 patients showed postoperative complications after gastrectomy. The OR of postoperative complications was 2.30 (95%CI: [1.78, 2.97]). According to the Clavien-Dindo grade (64), the PBT group had a higher incidence rate of grade Ⅲ-Ⅴcomplications (OR: 2.50, 95%CI: [1.71, 3.63]; p<0. 01), whereas no significant difference was seen in grade I-II (OR: 1.12, 95%CI: [0.63, 2.00]; p=0.69). (Table 4) The forest plot and funnel plot were shown in Figure 4 and Figure S4.  **Long-term Outcomes**  **Overall survival**  36 studies reported data on OS. Data on 5-year OS was available from 28 studies and HRs after multivariable analyses were extracted from 24 studies. The total number of enrolled patients was 25122, with individual samples ranging from 103 to 2884 (median 699). The HR of 5-year OS was 2. 39 (95% CI: [2.00, 2.84], P < 0.01) and the summary of the multivariable HR was 1. 43 (95% CI: [1. 24, 1.63]). Measure of heterogeneity indicates a high degree of variability about 5-year OS (HR: *I*^2^ = 83%, P < 0.01; multivariable HR: *I*^2^ = 74%, P < 0. 01). The random-effects model was used to obtain estimates.  **Disease-free survival**  17 studies reported data on DFS. Data on 5-year DFS were available from 16 studies and HRs after multivariable analyses were extracted from 9 studies. The 5-year DFS was lower in patients with PBT than NPBT patients. (HR= 2.26, 95% CI: [1.68, 3.05]; multivariable HR=1.44, 95% CI: [1.18, 1.75]). *I*^2^ as shown in Table 2. The funnel plot showed obvious asymmetry. (Figure S2)  **Disease-specific survival**  9 studies reported data on DSS. Data on 5-year DFS were available from 7 studies and HRs after multivariable analyses were extracted from 6 studies. The 5-year DFS was lower in patients with PBT than NPBT patients. (HR= 2.23, 95% CI: [1.35, 3.70]; multivariable HR= 1.35, 95% CI: [1.21, 1.51]). *I*^2^ as shown in Table 2. The funnel plot showed obvious asymmetry. (Figure S3) |  |
|  | 20c | A stratified analysis of OS was performed and the results were shown in Table 3. Publication years (before or after 2010), NOS score (≤7 stars or >7 stars), geographical location (west or east), average age (≤60 or >60), EBL (≤500ml or >500ml), PBT trigger (Hb<7g/L or Hb<8g/L), PBT rate (≤40% or >40%) and quantity (4U≤50% or 4U>50%) did not change the outcome significantly, which showed the result was robust. (Table 3) |  |
|  | 20d | Sensitivity analysis, which explored the effect on overall results by sequentially omitting individual studies, and a baujat plot was conducted to explore the source of heterogeneity between studies. (Figure S5-S6). |  |
| Reporting biases | 21 | 6 studies might be the main reason for the high heterogeneity. The funnel plot showed obvious asymmetry and publication bias was detected. (Figure S1) |  |
| Certainty of evidence | 22 | Stratified analysis and sensitivity analysis did not change the outcome significantly, which showed the result was robust. |  |
| **DISCUSSION** | | |  |
| Discussion | 23a | To date, the effects of PBT on the prognosis of gastric cancer patients undergoing gastrectomy were still controversial, and consensus had not yet been reached finally. The review and meta-analysis involved 51 studies with 41864 gastric cancer patients. To our best knowledge, this analysis represented the largest assessment of current research that targeted the impact of PBT on the long- and short-term outcomes. A primary finding was that PBT was associated with worse prognosis than the NPBT group.  Specificly, the results of the meta-analysis showed that PBT was associated with worse 1-,2-,3-and 5-year OS (82% vs 91%; 66% vs 80%; 57% vs 72%; 47% vs 65%), DFS (76% vs 88%; 61% vs 76%; 53% vs 74%; 52% vs 73%), and DSS (86% vs 89%; 64% vs 74%; 53% vs 66%; 48% vs 64%). The results were similar to the conclusions of previous research (55, 65-68). Similar results were found in other meta-analyses of other solid cancer, including colorectal cancer, hepatic cancer, esophageal cancer, and pancreatic cancer. Further, we conducted stratified analysis and sensitivity analysis of OS and the results were consistent and credible. The mechanism could be partially attributed to the suppression of the immune system induced by blood transfusion. Firstly, Some studies showed that the patients with previous blood transfusions experienced changes in the immune system involving inhibition of T cells and alteration in T cell subsets. Secondly, transfusion could trigger a series of a cascade of the immune system, including inhibition of the immunoregulatory cytokine IL-2, and the release of immunosuppressive prostaglandins. Besides, blood transfusion could induce transfusion-related immunomodulation (TRIM), further inhibits the function of macrophages and monocyte, and might lead to the decline of immune surveillance and enhance the potential for tumor growth and cellular metastasis.  Significant differences in the clinicopathological characteristics were found between the PBT group and NPBT group, which were consistent with previous studies . Compared with the NPBT group, the PBT group was more likely to be anemic and had lower Hb levels. Previous studies had shown that preoperative anemia was a powerful predictor of the need for blood transfusion and independently associated with an increased risk of mortality in patients undergoing surgery, even to a mild degree. Besides, the PBT group had more advanced tumor stages, more open surgery or total gastrectomy, and more EBL. Intraoperative blood transfusion was more likely to result from the complicated operation, especially large EBL. In addition, patients with transfusion were older and had more comorbidities, which might also be one of the important reasons for the poor prognosis in the PBT group.  Moreover, our findings showed that the PBT group had a higher postoperative complication rate. After grading the complications according to the Clavien-Dindo grade system, PBT was particularly related to grade Ⅲ-Ⅴ complications, but there was no significant difference in grade Ⅰ-Ⅱ when compared with the NPBT group. To date, the mechanisms that targeted the association between PBT and postoperative complications were unclear. Previous studies showed that the clinicopathological features of the patients in two groups might independently influence the postoperative complications. Compared with the NPBT group, patients with PBT were prone to suffer from more surgical trauma and had less tolerance for surgery because of their poor clinical condition. These clinicopathological factors, including old, advanced tumor stage, and complicated type of surgery, might be also associated with postoperative complications. Relevant mechanisms were expected to be demonstrated further. |  |
|  | 23b | For obvious ethical reasons, no randomized controlled trial (RCT) was searched and included in this meta-analysis. |  |
|  | 23c | The heterogeneity of some results was high in this meta-analysis, which might be attributed to the wide span of publication years, different transfusion triggers, and lacking PBT guideline. In addition, few studies presented the data on the amount and components of blood transfusion and the time of PBT, this meta-analysis failed to conduct further research. |  |
|  | 23d | More research was expected to explore the role of PBT and the appropriate PBT management strategy. |  |
| **OTHER INFORMATION** | | |  |
| Registration and protocol | 24a | This meta-analysis was performed according to the PRISMA Checklist. The protocol has been registered in the International prospective register of systematic reviews database (Prospro number: CRD42022314772) |  |
|  | 24b | [https://www. crd. york. ac. uk/PROSPERO/](https://www.crd.york.ac.uk/PROSPERO/). |  |
|  | 24c | According to the follow-up research, the proposal was modified appropriately |  |
| Support | 25 | This study was funded by the National Key R&D Program of China (Grant No. 2017YFC0908300). |  |
| Competing interests | 26 | The authors have declared no conflicts of interest. |  |
| Availability of data, code and other materials | 27 | All data were obtained from corresponding studies. All included studies are available from an online database. |  |
